# Increasing autobiographical memory specificity: Using kindness meditation to impact features of memory retrieval

**DOI:** 10.1371/journal.pone.0287007

**Published:** 2023-06-28

**Authors:** Amanda Lathan, Barbara Dritschel

**Affiliations:** School of Psychology and Neuroscience, University of St Andrews, St Andrews, United Kingdom; University of Bologna: Universita di Bologna, ITALY

## Abstract

Individuals with a history of depression have an increased risk for future episodes. This risk has been linked with impaired features of autobiographical memory retrieval that remain when depressive symptoms abate, including memory specificity, remoteness, valence, and vantage perspective. Rumination has been shown to influence these impairments and can be reduced via compassion training. We therefore investigated the effects of a self-compassion meditation on autobiographical memory retrieval in remitted depression. Baseline data were collected (*n* = 50) using an extended version of the Autobiographical Memory Test where participants with remitted depression retrieved specific memories from a remote time period (10 cues) and from any time period (10 cues). Valence and vantage perspective were rated. Participants were then randomly allocated to a self-compassion meditation or (control) colouring intervention group. Baseline measures were reassessed after four weeks of the intervention. Results revealed increased retrieval of specific memories in the self-compassion group in comparison to the colouring group, and an increase in positive and field memories across groups while no remoteness changes were observed. This self-compassion meditation demonstrated initial promise as an intervention to influence features of autobiographical memory retrieval in remitted depression. Improvements were shown in specificity, valence, and vantage perspective. Addressing these features with this type of intervention might reduce a cognitive vulnerability to depression and should be investigated in future studies.

## Introduction

Depression is characterised by somatic symptoms such as poor sleep, low energy, and psychomotor disturbances [1] as well as impairments to behavioral and cognitive domains. For example, facial emotion recognition has been shown to be impaired in depression, where recognition accuracy of happy facial expressions of emotion is reduced [2]. Further, emotions have been observed to impact response inhibitions which allow us to suppress inappropriate actions [3, 4]. In depression, medium to large performance impairments have been revealed on response inhibition tasks such as the Stroop colour-word task [5]. Furthermore, behavioral impairments in selective, sustained, and divided attention have been observed in depression [6], in addition to motor control deficits [7]. This collection of findings suggests that impairments in emotion regulation may impair cognitive processing in depression and that targeting emotion regulation may be an important strategy for improving it. The current paper focuses on one particular cognitive process shown to be disrupted by depression, the retrieval of autobiographical memories, our memory for personal life experiences [for a review, 8]. Autobiographical memory (AM) is essential to human functioning in areas such as self-concept [9], emotion regulation [10], and problem-solving [11, 12]. While retrieval impairments have been widely studied in currently depressed samples [13], there is a paucity of research in remitted samples. Examination of AM retrieval in remitted depression is imperative to help identify risk factors for future episodes and underlying mechanisms of depression that can inform preventive and treatment interventions. In particular, the current study is interested in how maladaptive AM retrieval styles may manifest in remitted depression following a self-discrepancy induction designed to induce cognitive reactivity. Further we extend research by looking at the impact of a loving-kindness meditation intervention on the features of AM retrieval in remitted depression following this induction. Loving-kindness meditation involves practicing unconditional kindness to oneself and others. Encouraging greater kindness to the self could reduce the impact of self-discrepancies on AM retrieval by reducing the negative affect associated with these judgements and their impact on AM retrieval. One AM retrieval difficulty in remitted depression is specificity, the ability to retrieve highly contextualized events. Specific memories refer to events that occurred at a particular time and place over the course of one day (e.g. my graduation from university) whereas overgeneral memories (OGM) are comprised of extended (e.g. my holiday to Greece) and categoric memories (e.g. my walks in the park on Fridays). Both current and remitted depressed individuals have difficulty retrieving specific memories of personal events [for a review, 14]. Instead, when asked to retrieve such memories, OGM are often produced which are more abstract as they describe general features of repeated events.

A key mechanism that is linked to OGM is rumination [15, 16], an abstract style of repetitive thinking grounded in discrepancies of personal characteristics that further elaborates conceptual information. Rumination disrupts the search for specific memories because it increases the likelihood of being captured by general knowledge when trying to retrieve specific memories. There is robust evidence for a positive association between rumination and OGM in current depression [17]. However, this association has been inconsistently reported for non-clinical samples including remitted depression [18]. One possible explanation is that rumination has been predominantly assessed through a trait measure suggesting trait rumination may not be sufficient to consistently activate OGM in remitted depression. Raes et al. (2012) therefore proposed that if state rumination was induced it might result in increased OGM. To investigate this proposal, Raes et al. (2012) administered the AMT to individuals with remitted depression, then used a self-discrepancy induction (that highlighted the difference between the actual and ideal self) to elicit state rumination, and subsequently administered the AMT again. Prior to the self-discrepancy induction, no association was found between trait rumination and OGM. However, after the induction a positive association was revealed in high trait-ruminators, suggesting that higher state rumination might be essential to this mechanism’s effects. Further, findings highlighted the context-dependent activation of OGM and suggested that OGM is a form of depressogenic cognitive processing, that increases when cognitive reactivity is induced. Broadly, cognitive reactivity occurs when negative cognitive processing (e.g. dysfunctional attitudes, negative thinking patterns) is activated by stressful situations [19]. Importantly however, Raes et al. (2012) did not examine the effects of activated self-discrepancies on additional features of AM retrieval such as remoteness, valence, and vantage perspective for instance. An examination of these factors would provide a more comprehensive insight into the impact of cognitive reactivity on AM retrieval in remitted depression.

Remoteness of memories (the age of the memory’s event) has been suggested to influence several characteristics of AM and therefore an important question is whether remoteness would impact AM retrieval in remitted depression following a self-discrepancy induction. Falco et al. (2015) required dysphoric and non-dysphoric individuals to complete a variant of the AMT and date the memory after retrieval [20]. Results revealed a positive correlation between retrieval of more remote memories and dysphoria. Further, OGM was related to remoteness regardless of dysphoria, suggesting that more remote memories tend to be less specific. As the Falco et al. (2015) study focused only on dysphoric individuals, it is unclear whether a tendency to retrieve more remote memories exists in remitted depressed samples. If the tendency to show greater OGM for remote memories is a trait of individuals prone to depression, for instance, it should occur in both current and remitted depression and might be more pronounced when a focus on self-discrepancies is activated (i.e. an instance of cognitive reactivity). For example, remote memories might be used to distance oneself from recent memories that painfully highlight their current discrepancies, particularly as they tend to be overgeneral [20].

Memory valence has been found to be associated with remoteness of specific memories [21]. Kim et al. (2018) demonstrated that positive-specific memories were more remote than negative-specific memories in individuals with Major Depressive Disorder (MDD) compared with controls, suggesting that remoteness of specific memories may depend upon valence in a currently depressed sample. Retrieving more remote-positive memories might contribute to a negative self-concept given the role of recent memories in constructing self-concepts [21]; however, it is unclear whether this retrieval tendency is prevalent in remitted depressed samples following a self-discrepancy induction. Positive memories might be more remote if they are discrepant with the current self. This is in line with the self-memory system, which postulates that specific memories should be non-discrepant with one’s actual (current) self-image and goals [9]. Thus, if a positive cue word triggers actual-ideal self-discrepancies, a more remote search might be activated to distance oneself from discrepant memories and maintain self-coherence.

Remote memories are also associated with memories being recalled from an observer perspective (third-person perspective), in comparison to a field perspective (first-person perspective). Field memories often contain the emotions the individual experienced during the original event [22], and thus contain more emotionality [23], are more vivid, and are more recent [24]. In comparison to healthy controls, depressed and remitted depressed individuals [25] tend to retrieve a greater proportion of categoric-observer memories than specific-field memories on the AMT. This finding is corroborated by literature that has demonstrated the tendency for remote memories to be more observer memories [26] and for dysphoric samples to retrieve more remote-overgeneral memories [20].

Moreover, emotional valence of the memory has been found to be associated with vantage perspective. While healthy controls tend to use a field perspective for the recall of positive AM [27, 28], depressed and remitted samples display an observer perspective for positive AM [29]. Given observer memories have been associated with less emotionality [24], positive-observer memories might not repair mood in the same way as positive-field memories [30]. It is therefore possible that activated self-discrepancies could influence vantage perspective and positive memory retrieval. When self-discrepancies are activated, observer perspective might be used to lessen the emotionality (e.g. pain) of memories where one’s actual self in the memory does not align with their ideal self.

Given activated self-discrepancies might affect features of AM retrieval, such as specificity, remoteness, valence, and vantage perspective, it is prudent to examine methods to reduce self-discrepant thinking. For instance, loving-kindness meditation (LKM) might reduce self-discrepant thinking by augmenting self-compassion. Three main attributes of self-compassion have been identified: self-kindness, common humanity, and mindfulness, an awareness of present moment suffering without the use of avoidance [31]. Given the attribute of self-kindness, LKM might lessen self-discrepancies by reducing a negative focus on the self as LKM employs a mantra of self-kindness. The self-kindness attribute has been shown to encourage kindness towards oneself and mitigate the impact of negative self-judgment when self-failures are perceived [32].

**Fig 1:**
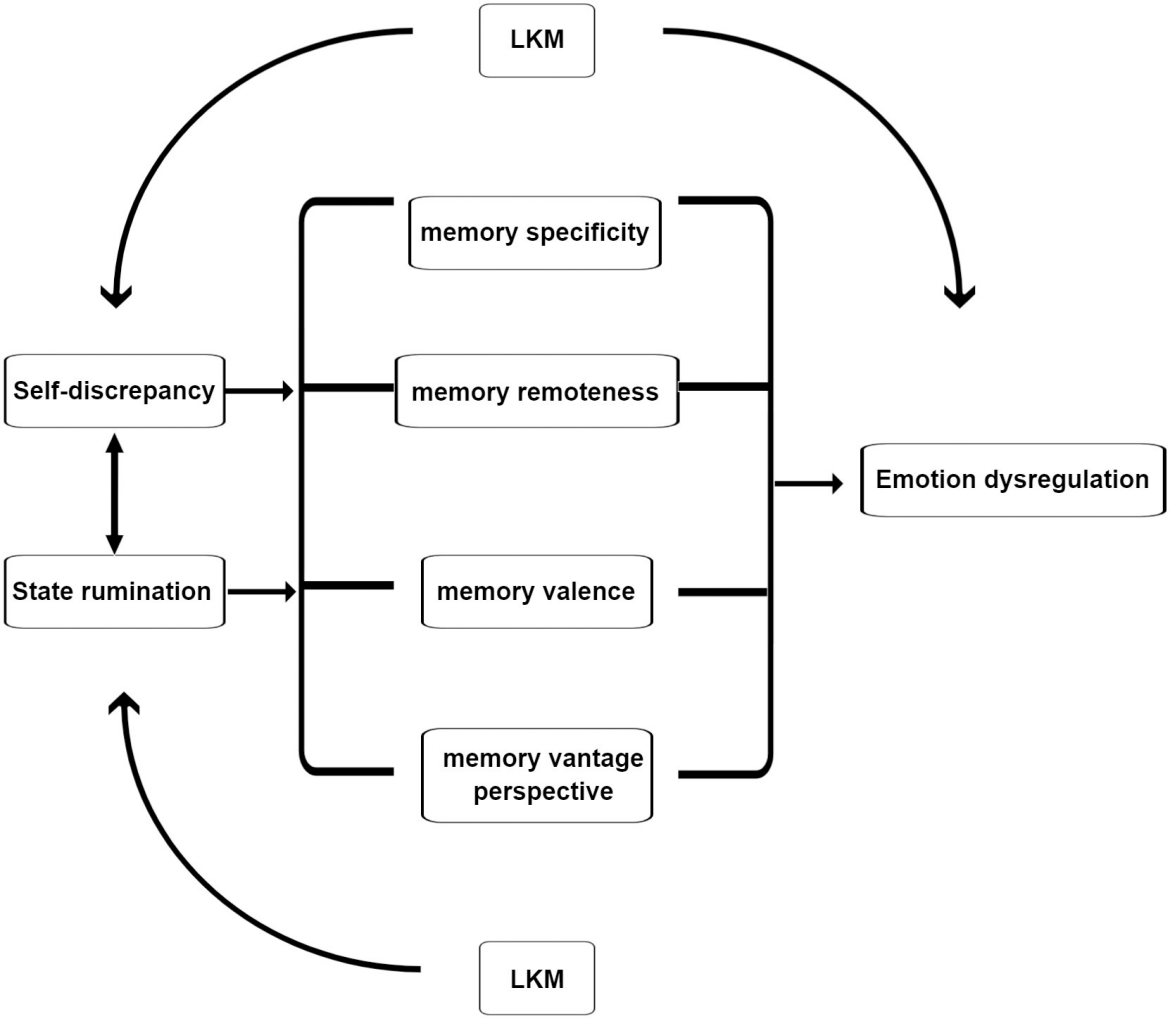
Outline of proposed mechanisms by which features of AM retrieval come to be compromised.

A further focus of LKM is on extending compassion towards others. This aspect of the meditation might improve a sense of common humanity which might help one to feel less alone in their pain and shift their focus away from self-discrepant thinking that focuses solely on the self. For example, one study compared the effects of a compassion training programme (Compassion Cultivation Training) with mindfulness-based stress reduction where participants engaged in 2.5-hours of face-to-face sessions per week and 30–45 minutes of daily practices [33]. In comparison to the mindfulness training, the compassion training achieved improvements in rumination, thought suppression, stress, anxiety, and depression via changes in socio-emotional mechanisms such as common-humanity and empathy [33]. Although studies have been conducted examining the impact of compassion training on rumination, a cognitive thinking style associated with depression, this is the first study to examine its impact on AM retrieval which is the repository for information about the self. LKM might increase the focus on common-humanity and empathy (socio-emotional mechanisms), for example, as demonstrated with Compassion Cultivation Training [33] which could shift a focus away from self-discrepant thinking, influencing the retrieval of AM as shown in Fig 1. For example, one component of LKM removes the focus from self, and thus possibly self-discrepant thinking, by encouraging visualisation of others. An fMRI study showed that meditators who practiced LKM had reduced functional connectivity between nodes of the default mode network that are argued to be involved in self-referential processing [34]. Moreover, during retrieval of specific memories, in comparison to never depressed participants, those with current and remitted depression have shown enhanced activation in regions of the brain involved in self-referential processing (e.g. medial frontal gyrus, precuneus) [35].

Rumination about self-relevant, semantically related information, and self-discrepant thinking are proposed to compromise the search for a specific memory and influence the remoteness, valence, and vantage perspective of the memory. Difficulty recalling specific memories, biases for retrieving more remote memories, using the observer perspective, and recalling negative memories then leads to emotion dysregulation, for example. LKM is proposed to reduce state rumination, self-discrepant thinking, and emotion dysregulation, thereby influencing features of AM retrieval.

Kindness based practices, such as LKM, have further been shown to enhance emotion regulation and higher-level perspective-taking [36], which could also remove the focus from self-discrepant thinking, impacting the retrieval of AM. Reducing self-discrepancies might change negative self-schemata to have a more positive view of self. It could be that LKM aids in the cultivation of positive self-schemata by targeting emotion regulation. For example, LKM training has been shown to augment positive affect [37] which might activate more positive self-schemata. Further, LKM has been shown to reduce heart rate and skin conductance and increase heart rate variability in comparison to a positive-excitement condition [38]. This pattern of reduced arousal and increased parasympathetic activation is associated with effective emotion regulation during adverse events.

LKM might also impact OGM through its impact on neurobiological mechanisms underpinning OGM. For example, low tryptophan (an essential amino acid required for the production of serotonin) has been identified as a possible neurobiological mechanism underlying OGM as low-dose tryptophan depletion reduced output of specific AM when remitted depressed women were asked to retrieve specific AM to negative cue words [39]. Further, differences between those with and without depression in the blood levels of molecules such as kynurenic acid (a product of normal metabolism of tryptophan) might be responsible for lower grey matter volumes in the hippocampus and precuneus in those with depression. These effects have been associated with reduced recall of specific AM [35].

Tryptophan’s primary catabolic route has been identified to be the kynurenine system [40]. Activation of the kynurenine system is conducive to the development of neuropsychiatric disorders such as depression [41]. Moreover, the enzymes of the tryptophan-kynurenine system are activated by inflammation [41] which has been associated with depression [42]. Importantly, compassion-based meditations have been shown to reduce inflammation [43]. Inflammation is further associated with greater cortisol which is associated with reduced AM specificity [44]. Administration of a glucocorticoid antagonist reduced cortisol in hippocampus receptors and resulted in greater AM specificity in healthy controls than a placebo [45]. Further, mitochondrial (cell organelle) dysfunction has been found to contribute to the development of depression [41]. Mitochondrial dysfunctions have been shown to cause attrition of telomeres (caps at the end of chromosomes) [46]. This attrition is associated with worse health [47] and greater depression [48]. Importantly, LKM practitioners have been shown to have longer relative telomere length than non-meditators at trend level and significantly longer relative telomere length in comparison to controls among women [49]. Furthermore, Zen meditators have been found to have longer median telomere length in comparison to non-meditators, where age, absence of experiential avoidance, and common humanity (a component of self-compassion) were significant contributors of telomere maintenance, highlighting self-compassion as a critical factor in biological benefits of meditation [50].

We therefore used two groups with remitted depression to explore whether a brief LKM could influence AM specificity, remoteness, valence, and vantage perspective by reducing self-discrepancies. LKM is broadly accessible in that individuals do not need to leave their home to practice, and the meditation can be completed within ten minutes. An active control colouring group was also used to control for the possibility that effects of LKM might be due to distraction. Distraction has been shown to effectively reduce sad mood in both current and remitted depressed samples [30]. It has been suggested that distraction may be more preventative in engagement with mood-congruent thinking that might otherwise maintain negative moods [51].

In a remitted depressed sample, we used positive cue words in the AMT with an aim to examine the effects of LKM on four features of AM retrieval: specificity, remoteness, valence, and vantage perspective. We aimed to increase retrieval of specific, positive, and field memories in remitted depression after a four-week LKM intervention. Positive cue words were used with an intention to elicit a greater magnitude of impact of self-discrepancies and because vantage perspective research has indicated a greater influence on positive memories [29]. We further aimed to identify whether a remitted depressed sample would display more remote as opposed to recent memory retrieval (at baseline) as previously shown in dysphoria [20]. Given the significant impact of remoteness on OGM [20] and that OGM is predictive of future episodes of depression [52], remoteness is important to examine in remitted depression. The current study employed a manipulation of time in which participants were required to retrieve half the memories from more than one year ago (remote condition) and half the memories from any time period (free-recall condition). This manipulation was also employed by Falco et al. (2015) [20]. Additionally, we investigated whether positive-specific memories would be retrieved more remotely than recently (at baseline) in remitted depression as observed in current depression [21].

It was hypothesised that in comparison to the colouring group, the LKM group would show an increase in AM specificity across both time conditions (free-recall and remote conditions). Moreover, an interaction was expected where specificity would increase more in remote memories in the LKM group. Literature has demonstrated that remote memories tend to be more overgeneral than recent memories [20], suggesting that there might be a greater opportunity to improve specificity in remote memories. We further predicted that in comparison to controls, the LKM group would retrieve more positive-specific memories across both time conditions. An interaction was also expected where positive-specific memory retrieval would increase more in recent memories (free-recall condition) in the LKM group given positive memories might be more remote if they are discrepant with the current self [9], and self-discrepancies are predicted to decrease in the LKM group. We also predicted that in comparison to controls, the LKM group would retrieve more specific-field memories across both time conditions. Moreover, an interaction was expected where the use of field perspective would increase more for positive-specific memories (across both time conditions) in the LKM group. It might be that activated self-discrepancies increase the use of observer perspective for positive memories and self-discrepancies would be predicted to decrease in the LKM group. We further predicted that in both groups at baseline, more remote memories would be retrieved than recent memories (free-recall condition) and positive-specific memories would be retrieved more remotely than recently (free-recall condition).

## Methods

### Participants

*A priori* power analyses were conducted to estimate the required sample size for the change in AM retrieval features per group. Calculations indicated that 25 participants per group would provide 90% power (α = 0.05) for repeated measures Analysis of Variance (ANOVAs). In total, 55 students with remitted depression from the University of St Andrews were voluntarily recruited. After starting the study, five participants discontinued the treatment intervention (4 LKM, 1 colouring) and withdrew from the study. Complete data were therefore available for 50 participants. Thirty-four participants identified as female, 14 as male, and 2 as non-binary. Participants had a mean age of 21.68 (SD = 5.01, range 18–42). Seven participants (14%) had a history of 2 episodes of depression, 9 participants (18%) had experienced 3 episodes, 31 (62%) reported 4 or more episodes, and 3 participants (6%) reported their depressive experience as “continual” (however, these participants met diagnostic criteria for remitted depression). Participants were allocated to the LKM (*n =* 25) and colouring (*n =* 25) groups using Simple Randomisation, where a randomised list was generated via Random Allocation Software [53]. Groups did not differ significantly in gender distribution, (*t*(48) = -0.54, *p* = 0.59, *d* = -0.15, BF10 = 0.32), age, (*t*(48) = -0.06, *p* = 0.96, *d* = -0.02, BF10 = 0.28), or the number of previous MDD episodes, (*t*(48) = -0.45, *p* = 0.66, *d* = -0.13, BF10 = 0.31). However, no reliable conclusion could be drawn regarding group differences in age of MDD onset, (*t*(48) = 0.78, *p* = 0.44, *d* = 0.22, BF10 = 0.36), or intervention compliance rate, (*t*(48) = -1.56, *p* = 0.13, *d* = -0.44, BF10 = 0.76). Demographic and diagnostic data are reported in Table 1 per group.

**Table 1:** Group demographic, diagnostic, and intervention compliance data.

Demographic Information	LKM	Colouring
Current meditation practice, *n*	9	8
Current self-compassion practice, *n*	3	4
Compliance rate, M *percentage* (SD)	61.37 (22.75)	70.91 (20.41)
Previous MDD episodes, M (SD)	6.27 (5.55)	6.96 (4.99)
Age of MDD onset, M (SD)	16.26 (3.63)	15.50 (3.29)
MDD with melancholic features, *n*	0	0
Dysthymia, *n*	0	0
Hypomanic episode, *n*	0	0
Manic episode, *n*	0	0
Panic disorder (current), *n*	4	1
Panic disorder (limited symptoms), *n*	10	4
PTSD (current), *n*	0	0
Alcohol dependence (current), *n*	3	3
Alcohol abuse (current), *n*	1	0
Alcohol dependence (lifetime), *n*	4	4
Alcohol abuse (lifetime), *n*	1	1
Generalized anxiety disorder (current), *n n*	5	10

Diagnoses were assessed via the MINI-Plus [54]. Compliance was operationalized as daily intervention engagement throughout the intervention. Thus, a participant who meditated or coloured 20 out of 28 days would demonstrate 71.43% compliance rate.

Participants aged 18–45 were recruited via university communications where individuals with a previous experience of depression were invited to participate in a study that involved either a meditation or colouring practice for four weeks. Participants were included in the study if they met criteria for past episodes of Major Depression but not a current episode as assessed via the MINI-Plus [54] by a trained interviewer. Inter-rater reliability was completed for 20% of MINI-Plus interviews by a trained interviewer, and 100% agreement was observed between raters on all diagnoses. All participants had a history of two or more episodes of depression. Participants with comorbidities were included in the study with the exception of those with bipolar disorder. The study received ethical approval from the School of Psychology and Neuroscience Ethics Committee and participants received monetary compensation.

### Measures

At baseline, the first six participants completed all questionnaires in writing, while the AMT was administered via Microsoft Excel [55], as described below. At follow-up, due to the Covid-19 pandemic, the research was conducted online using Microsoft Teams to create a setting similar to an *in vivo* setting. While on Microsoft Teams, Qualtrics (Provo, UT) was used for all measurements. For the remaining participants, all parts of the study were conducted via Microsoft Teams and Qualtrics. Bayesian independent samples t-tests revealed no reliable conclusion could be drawn regarding differences in the means of AM retrieval data between responses via Excel or Qualtrics: specificity, (*t*(48) = 0.06, *p* = 0.95, *d* = 0.03, B10 = 0.39), total positive-specific memories, (*t*(48) = 0.21, *p* = 0.84, *d* = 0.09, BF10 = 0.40), total field-specific memories, (*t*(48) = -0.14, *p* = 0.89, *d* = -0.06, BF10 = 0.39).

#### MINI-Plus structured interview

The MINI-Plus [54] was used to assess diagnostic status at entry into the study. The MINI-Plus is a structured diagnostic interview that was developed for quick and accurate diagnoses of psychiatric disorders according to the DSM-V criteria. The interview has been shown to have high inter-rater reliability and good sensitivity [56]. It has been further demonstrated to have good specificity with the ability to exclude patients who do not have the disorder [56]. The current study revealed 100% agreement between the interviewer and second rater.

#### Depression, Anxiety, Stress Scales (DASS)

The DASS [57] is a set of three self-report scales within a 42-item questionnaire designed to measure the magnitude of an individual’s depression, anxiety, and stress symptoms. Items are rated on a 4-point scale (*Did not apply to me at all* to *Applied to me very much*, *or most of the time*) and instructions indicate that participants should choose the statements that reflect how they have been feeling over the past week. Higher scores indicate higher symptom severity. The DASS has been shown to have typically high internal reliability and the DASS depression scale has been highly correlated with Beck’s Depression Inventory [58] while the DASS anxiety scale has been highly correlated with Beck’s Anxiety Inventory [59, 60]. Internal consistency in this sample was high, Cronbach’s α = 0.94.

#### Rumination Response Scale (RRS)

The RRS [61] is a 22-item self-report questionnaire intended to assess trait rumination as the extent to which individuals respond to depressed mood by focusing on its causes and implications. Items are rated on a 4-point scale (*Almost never* to *Almost Always)* and instructions indicate that statements should be chosen based on what the individual “generally does”. The brooding and reflection subscales consist of five items each [62] and the RRS has been shown to have reliability and discriminant validity of the subscales [61]. Internal consistency in this sample was good, Cronbach’s α = 0.88.

#### Self-Compassion Scale (SCS)

The SCS [31] is a 26-item self-report questionnaire developed to assess trait levels of self-compassion via explicit representations of thoughts, emotions, and behaviors associated with the questionnaire’s components [63]. Items are rated on a 5-point scale (*Almost never* to *Almost Always)* and instructions indicate that statements should be chosen based on how often you behave in the stated manner. The SCS has been shown to have consistently high internal reliability across a wide variety of populations [63, 64]. Internal consistency in this sample was high, Cronbach’s α = 0.90.

#### Self-discrepancy induction

The self-discrepancy induction is a 50-item self-report questionnaire designed to induce a focus on self-discrepancies and elicit state-rumination [18, 65]. Items include 50 positive single-word characteristics (e.g. attentive) that are different from the AMT cue words. Participants evaluated each word on two dimensions; they were instructed to carefully think about the characteristic and rate i) their ideal self: the extent to which they would like to possess the characteristic and ii) their actual self: the extent to which they currently possess the characteristic. Scales range from 0 (*not at all*) to 10 (*very much*).

#### Visual Analogue Scales (VAS)

Three visual analogue scales were used: mood, experienced discrepancy, and focused discrepancy in line with previous research [18]. Instructions indicate that individuals should respond according to their current state. The scales were administered both before and after the self-discrepancy induction to assess changes in current mood and self-discrepancies following the induction.

#### Visual analogue mood scale

Participants were asked to rate their current level of sadness (0 –*not at all sad*, 100 –*very sad*). This VAS has been shown to have high reliability and validity [66] and has been highly correlated with Beck’s Depression Inventory [58, 60].

#### Visual analogue experienced self-discrepancy scale

Participants were asked to rate the extent to which they currently experience a discrepancy between their actual and ideal self (0 –*not at all a difference*, 100 –*a huge difference*).

#### Visual analogue self-discrepancy focus scale

Participants were asked to rate the extent to which their current thoughts were about wondering how large the difference is between their actual and ideal self (0 –*my thoughts have absolutely nothing to do with it*, 100 –*my thoughts have a lot to do with it*).

#### Autobiographical Memory Test (AMT): Extended version

The AMT [67] measures memory specificity by presenting cue words to participants and asking for autobiographical memories to be provided. In clinical populations, the AMT is often presented orally [21, 24]; however, prior studies have also successfully employed written versions of the task [68]. The current study adopted a written, extended version of the task, which was presented twice, once at the baseline and once at the follow-up assessment. Two comparable versions of the task were used where each version contained 20 positive cue words matched for frequency [69, 70], emotionality [71–73], and imageability [74, 75].

Participants were instructed to retrieve a specific, personal memory in response to the cue word by typing it into the available text box via Qualtrics within 60 seconds. In Excel, the text box was an enlarged cell. A specific memory was defined as a memory of an event which lasted less than a day and occurred at a particular time and place. Examples of correct and incorrect responses were provided, and two practice trials were given at baseline before the task commenced. Participants proceeded to the test phase once they successfully completed the practice trials. In the test phase, participants were requested not to repeat memories. Additionally, participants were instructed to recall a memory from any time (free recall condition) for half of the cues, whereas memories were requested to be from more than one year ago (remote condition) for the other half. Remoteness condition was randomised in presentation order. Further, cue words were randomised between remoteness conditions and in presentation.

After 60 seconds, the recall page automatically progressed in Qualtrics, whereas if completed via Excel, the researcher’s timer sounded to manually continue to the next sheet. On the subsequent page, participants were instructed to select answers to the following questions: i) Was the memory: positive, negative, neutral, or other? ii) From what perspective did you recall the memory: first-person, observer, blended, or no image? The blended option was described to include at least two of the perspective options. iii) When did the event occur? Participants were asked to provide a date of the memory’s event. Unlimited time was allowed to answer these questions.

Following previous studies [76], memories were coded as *specific* (lasting less than one day), *categoric* (summarised events), *extended* (lasting longer than one day), and *omission* (no response). One researcher scored all memories and a sub-sample of 280 memories was rated by a second assessor blind to the treatment condition to establish reliability of coding. The weighted kappa for the distinction between specific and non-specific responses was good, k = 0.85 (95% agreement). Analyses reported were based on the number of each memory type retrieved.

#### Experimental and control treatment interventions

Groups were standardized in procedure and adherence was uncontrolled but monitored. Participants of both groups were encouraged to continue with any current medication or other treatment phase as they would have done otherwise. Both groups completed their respective daily practices at home, individually, for four weeks.

Upon completion of baseline measurements, participants in the LKM group were taught the meditation by a certified instructor via Microsoft Teams. LKM participants then independently practiced the meditation every day from either an upright, seated, or lying down position [77, 78]. Participants were informed that the meditation could be practiced at any time of day. Throughout the ten-minute meditation, different types of individuals were visualised separately (self, someone you find it easy to love, a stranger, someone you find it difficult to love, and everyone from the practice) for two minutes each, and the participant used the mantra “may I be happy, may I be healthy, may I be filled with loving-kindness and peace”. Participants were instructed to use a university learning platform to access recordings of the meditation every day (Moodle) [79, 80]. Two recordings were available for participants to choose at their own discretion: i) a guided LKM from a certified instructor where each section was introduced and an indication was provided when it was time to proceed to the next section, ii) a recording of gongs and silence, where the gongs occurred every two minutes to indicate it was time to proceed to the next section. These recordings were accessed via Moodle both as a measure of control and a means to capture compliance data. Participant meditation engagement was recorded, providing the date, time, duration, and chosen recording (guided or gongs) for every meditation.

In the control colouring group, participants were asked to (digitally) colour one mandala image of their choice per day. Digital colouring has shown a similar decrease in anxiety symptoms in comparison to pen-and-paper colouring [81], thus, participants used the free app, Coloring Book for iOS (*n* = 17) [82] or Free Mandala Coloring Book for Android (*n* = 8) [83] with both apps offering similar mandala images. While there was not a prescribed amount of time in which to complete the mandala, it took participants an average of 9.33 minutes to colour one image (SD = 5.13) and colouring was completed within one sitting at any time of day. The completed image was emailed to the researcher every day to monitor compliance.

### Procedure

The study was administered online via Microsoft Teams and Qualtrics (Provo, UT). Participants attended an initial session during which they provided informed, written consent, demographic information, and were interviewed using the MINI-Plus for screening and diagnostic purposes. The first session lasted an average of 45 minutes. Eligible participants were invited to a second session (within one week) during which they completed baseline questionnaires and the AMT extended version. Measurements were completed in the following order: i) DASS, ii) RRS, iii) SCS, iv) VAS, v) self-discrepancy induction, vi) VAS, vii) AMT extended version. Following completion of baseline measures, participants were randomly allocated by the lead researcher, using Simple Randomisation, to either the LKM or colouring group and were introduced to the treatment. A distraction task was subsequently administered that asked participants to spend three minutes thinking about neutral situations from a list of thirty (e.g. think about the colour of clouds). This second session lasted an average of 45 minutes. Within one week of treatment completion, participants were reassessed on all baseline measures and were then debriefed on the nature of the study. Participants in the colouring group were subsequently invited to learn the meditation and access the Moodle recordings. The final session lasted an average of 40 minutes.

### Statistical analysis

JASP (0.13.1) was used for statistical analyses and an alpha level of 0.05 was used for all statistical tests. The study’s design was between-subjects (LKM, colouring). The participant characteristics (e.g. age, number of previous MDD episodes, etc.) and baseline scores on the mood measures (e.g. DASS, RRS, etc.) were analysed using Bayesian independent samples t-tests. Bonferroni post hoc analyses were performed where appropriate for ANOVAs.

## Results

### Participants at baseline

The mean pre- and post-intervention scores for each group can be seen on Table 2. Bayesian independent samples t-tests revealed that no reliable conclusion could be drawn regarding group differences on DASS (*t*(48) = -0.94, *p* = 0.35, *d* = -0.27, BF10 = 0.41), RRS (*t*(48) = -1.15, *p* = 0.26, *d* = -0.33, BF10 = 0.49), or SCS (*t*(48) = -0.83, *p* = 0.41, *d* = -0.24, BF10 = 0.38) baseline measurements. The mean total DASS score across both groups at baseline was 33.38 (SD = 17.53; range: 2–73), while the DASS depression score was 12.60 (SD = 8.16; range: 0–35), indicating mild depressive symptoms on average. The mean baseline RRS score was 50.62 (SD = 10.96; range: 25–76), indicating moderate levels of rumination, while the mean SCS score was 2.69 (SD = 0.60; range: 1.69–4.31), indicating moderate levels of self-compassion.

**Table 2:** Means and standard deviations for relevant variables at pre- and post-intervention.

	LKM group		Colouring group	
Variable	Pre-intervention	Post-intervention	Pre-intervention	Post-intervention
	M (SD)	M (SD)	M (SD)	M (SD)
DASS total	31.04 (16.56)	27.16 (16.09)	35.72 (18.49)	29.52 (15.23)
DASS depression	11.24 (7.41)	9.24 (6.54)	13.96 (9.79)	11.92 (7.90)
RRS	48.84 (11.33)	42.84 (9.42)	52.40 (10.51)	50.60 (10.58)
SCS	2.62 (0.55)	2.97 (0.76)	2.76 (0.65)	2.87 (0.64)
VAS-mood	30.32 (20.09)	24.96 (21.22)	37.52 (20.67)	37.20 (22.91)
VAS-experienced	55.84 (24.97)	47.60 (21.83)	65.32 (26.44)	60.16 (23.15)
VAS-focused	56.04 (27.93)	48.92 (31.86)	65.56 (21.20)	51.92 (26.31)
Specific	13.20 (4.89)	15.32 (3.79)	11.92 (4.35)	11.80 (4.40)
Specific prop	66.00	76.60	59.60	59.00
Pos-specific	8.60 (3.30)	10.92 (3.76)	8.88 (3.27)	9.40 (4.84)
Pos-specific prop	43.00	54.60	44.40	47.00
Specific-field	8.20 (4.17)	10.28 (4.69)	7.24 (3.87)	7.92 (3.73)
Specific-field prop	41.00	51.40	36.20	39.60
Recent-specific	2.88 (2.01)	3.00 (2.06)	3.64 (2.25)	3.44 (2.36)
Recent-specific prop	28.80	30.00	36.40	34.40
Remote-specific	4.36 (2.52)	4.56 (2.62)	2.96 (2.67)	2.80 (2.50)
Remote-specific prop	43.60	45.60	29.60	28.00

Specific, positive (Pos-), and field memories include data regarding total mean numbers (across both remoteness conditions) while recent and remote specific include mean numbers from the free-recall condition. Mean proportions (prop) for specific, positive, and field data represent the mean number retrieved out of 20. Mean proportions for recent and remote specific data represent the mean number retrieved out of 10. Omissions were included in the denominator in line with previous research demonstrating that when formerly depressed participants made an omission, non-specific content was often retrieved [84]. Where data were missing, such as memory valence, the memory was excluded from corresponding analyses and removed from the proportion’s denominator. VAS scores reflect means post-self-discrepancy inductions.

### Manipulation checks at baseline

No reliable conclusion could be drawn regarding a significant change in sadness following the self-discrepancy induction at baseline, *t*(49) = -1.31, *p* = 0.20, *d* = -0.19, BF10 = 0.34. In line with Raes et al. (2012), given the self-discrepancy induction is not a sad mood induction, we did not expect a significant increase in sadness. Experienced self-discrepancy did increase significantly from pre- to post-induction, *t*(49) = -2.04, *p* = 0.05, *d* = -0.29, as did focused self-discrepancy, *t*(49) = -2.25, *p* = 0.03, *d* = -0.32, indicating the manipulation was successful.

### Autobiographical Memory Test

The descriptive statistics for the AMT variables pre- and post-intervention are shown in Table 2. As aforementioned, following previous literature [18, 84], we used the number of target memories (e.g. the number of specific memories) rather than the proportion of memories excluding omissions from the denominator. Crane et al. (2007) demonstrated that when formerly depressed participants made an omission, non-specific content was often retrieved, suggesting that removing omissions in the calculation of a specificity score, for example, might not reflect the most appropriate method.

### Correlations between rumination and memory specificity at baseline

After inducing state rumination, a significant, positive correlation (Pearson correlation coefficient) was observed at baseline between trait rumination (RRS) and the number of specific memories, *r*(50) = 0.45, *p* = 0.001, such that higher levels of trait rumination were associated with higher levels of specificity. Data were tested for outliers and linearity, meeting assumptions of the analysis. This correlation is in a surprising direction that contradicts previous findings [18].

### Autobiographical memory remoteness at baseline

To determine whether there was a tendency for more remote memories to be retrieved, we calculated the difference in number of recent-specific memories (up to one year old) versus remote-specific memories (over one year old) retrieved at baseline in the free-recall condition. Retrieval of recent-specific and remote-specific memories did not differ significantly, *t*(49) = -0.67, *p* = 0.51, *d* = -0.10, BF10 = 0.19, indicating that this tendency was not observed in remitted depression, contrary to our predictions. Given remote memories have been found to be more overgeneral however [20], it might be that the above analysis is not appropriate considering only specific memories were included. We therefore calculated the difference in number of recent memories versus remote memories retrieved at baseline regardless of specificity (i.e. including OGM) in the free-recall condition. Although it is difficult to assess the age of categoric memories given they do not concern one particular event, this analysis used the age of memories provided by participants. No significant difference was revealed however, *t*(49) = -0.13, *p* = 0.90, *d* = 0.02, BF10 = 0.16, suggesting that there was not a tendency for more remote retrieval in remitted depression.

We further measured the remoteness of specific memories by calculating a distance memory score (DMS) for each participant [20]. Remoteness was coded by assigning numerical value to the five categories of time we used: 1 corresponds to less than a week, 2 –a week to a month, 3 –a month to a year, 4–13 months to 5 years, and 5 –over five years. The DMS was calculated by using the mean numerical value for each specific memory, excluding overgeneral memories given the time period for categoric and extended memories is difficult to determine. The mean DMS across both groups in the free-recall condition at baseline was 3.27 (SD = 0.86), demonstrating that on average, specific memories were retrieved from one month to one year ago. This mean is similar to the DMS found in dysphoria [20]. The mean DMS across both groups in the remote condition at baseline was 4.25 (SD = 0.26), demonstrating that on average, specific memories in the remote condition were less than five years old (i.e. retrieved from 13 months to 5 years ago).

### Autobiographical memory valence at baseline

We calculated the difference in number of positive-specific recent memories (up to one year old) versus positive-specific remote memories (over one year old) retrieved at baseline in the free-recall condition. Contrary to expectations, no significant difference was revealed, *t*(49) = 0.99, *p* = 0.33, *d* = 0.14, BF10 = 0.24, suggesting that there is not a tendency for positive memories to be more remote in remitted depression.

### Changes in negative affect, rumination, self-compassion, and self-discrepancies, from pre- to post-intervention

Bayesian repeated measures ANOVAs were conducted on negative affect, rumination, self-discrepancies, and self-compassion with time (pre-and post-intervention) as a within-subjects factor, group (LKM, colouring) as a between-subjects factor, and intervention compliance as a covariate. An analysis of residuals confirmed the assumptions of linearity. For negative affect (total DASS), results were inconclusive regarding a main effect of time, BF10 = 2.40, and group, BF10 = 0.45, and there was no interaction between time and group, BF10 = 0.32. Results suggested no difference in the reduction of negative affect over time depending upon group. The Bayesian repeated measures ANOVA for depression (DASS depression) revealed inconclusive results regarding a main effect of time, BF10 = 0.94, and group, BF10 = 0.72, and there was no interaction between time and group, BF10 = 0.28, suggesting no difference in the reduction of depression over time depending upon group. Regarding rumination (RRS), there was a main effect of time, BF10 = 9.15, but inconclusive results regarding a main effect of group, BF10 = 1.93, and regarding an interaction between time and group, BF10 = 0.85. Results suggested no reliable conclusion could be drawn regarding a difference in the reduction of rumination over time depending upon group. Further, the Bayesian repeated measures ANOVA for self-compassion revealed inconclusive results regarding a main effect of time, BF10 = 1.00, while there was no main effect of group, BF10 = 0.006. Further, results were inconclusive regarding an interaction between time and group, BF10 = 1.18, suggesting no reliable conclusion could be drawn regarding a change in self-compassion over time depending upon group. There was a main effect of time for experienced self-discrepancies (*post*-induction from pre- to post-intervention), BF10 = 4.11, while results were inconclusive regarding a main effect of group, BF10 = 1.10, and regarding an interaction between time and group, BF10 = 0.41, indicating no reliable conclusion could be drawn regarding a difference in the reduction of experienced self-discrepancies over time depending upon group. Finally, there was a main effect of time for focused self-discrepancies (*post*-induction from pre- to post-intervention), BF10 = 3.83, while results were inconclusive regarding a main effect of group, BF10 = 0.43, and regarding an interaction between time and group, BF10 = 0.36, suggesting no reliable conclusion could be drawn regarding a difference in the reduction of focused self-discrepancies over time depending upon group.

### Changes in memory specificity from pre- to post-intervention

A repeated measures ANOVA was conducted on the total number of specific memories on the AMT (across both remoteness conditions) with time (pre- and post-intervention) as a within-subjects factor, and group (LKM, colouring) as a between-subjects factor. An analysis of residuals confirmed the assumptions of linearity. Results revealed a main effect of time, F(1, 48) = 4.73, *p* = 0.04, η_p_^2^ = 0.09, and group, F(1, 48) = 4.37, *p* = 0.04, η_p_^2^ = 0.08, and an interaction between time and group, F(1, 48) = 5.94, *p* = 0.02, η_p_^2^ = 0.11. Results demonstrated an increase in specificity over time depending upon group, where specificity significantly increased in the LKM group in comparison to the colouring group as hypothesised.

A Bayesian repeated measures ANOVA was then performed on the number of specific memories in the free-recall condition using remoteness (recent and remote) and time (pre- and post-intervention) as within-subjects factors, and group (LKM, colouring) as between-subjects factors. An analysis of residuals confirmed the assumptions of linearity. Results revealed no main effect of time, BF10 = 0.17, while results were inconclusive regarding a main effect of remoteness, BF10 = 0.64, and group, BF10 = 0.90. Results were further inconclusive regarding an interaction between time and group, BF10 = 0.99 and remoteness and group, BF10 = 1.75, while there was no interaction between time, remoteness, and group, BF10 = 0.14. Reliable conclusions could not be drawn regarding group differences in a change in specificity over time in the free-recall condition.

Moreover, to examine whether specificity increased over time in remote memories in the remote condition, a Bayesian repeated measures ANOVA was performed on the number of specific memories using time (pre- and post-intervention) as a within-subjects factor, and group (LKM, colouring) as a between-subjects factor. There was no main effect of time, BF10 = 0.23, while results were inconclusive regarding group, BF10 = 1.19, and regarding an interaction between time and group, BF10 = 0.84. Results demonstrated that specificity of remote memories (in the remote condition) was not significantly altered over time, while reliable conclusions could not be drawn regarding group differences in a change in retrieval of remote specific memories.

### Changes in positive memory retrieval from pre- to post-intervention

A Bayesian repeated measures ANOVA was conducted on the total number of positive-specific memories (across both remoteness conditions) with time (pre-and post-intervention) as a within-subjects factor, and group (LKM, colouring) as a between-subjects factor. An analysis of residuals confirmed the assumptions of linearity. There was a main effect of time, BF10 = 4.82, while results were inconclusive regarding a main effect of group, BF10 = 0.39, and regarding an interaction between time and group, BF10 = 1.05, contrary to our hypothesis (see Table 3 for memory valence means).

**Table 3:** Means and standard deviations for memory valence and vantage perspective at pre- and post-intervention.

	LKM group		Colouring group	
Variable	Pre-intervention	Post-intervention	Pre-intervention	Post-intervention
	M (SD)	M (SD)	M (SD)	M (SD)
Positive	8.60 (3.30)	10.92 (3.76)	8.89 (3.27)	9.40 (4.84)
Negative	1.60 (1.61)	0.84 (1.23)	1.08 (1.32)	0.64 (0.76)
Neutral	2.16 (2.36)	2.72 (2.03)	1.60 (1.56)	1.44 (1.56)
Other	0.64 (1.22)	0.76 (1.42)	0.36 (0.70)	0.12 (0.33)
Field	8.20 (4.17)	10.28 (4.69)	7.24 (3.87)	7.92 (3.73)
Observer	2.04 (2.39)	2.12 (2.13)	1.80 (1.38)	1.32 (1.82)
Blended	2.76 (2.82)	2.80 (2.92)	2.20 (2.27)	2.12 (1.99)
No image	0.12 (0.44)	0.20 (0.65)	0.64 (1.44)	0.12 (0.60)

Data include total mean numbers of specific memories (across both remoteness conditions).

A Bayesian repeated measures ANOVA was then conducted on the number of positive-specific memories in the free-recall condition using remoteness (recent and remote) and time (pre- and post-intervention) as within-subjects factors, and group (LKM, colouring) as a between-subjects factor. There was no main effect of time, BF10 = 0.18, remoteness, BF10 = 0.27, or group, BF10 = 0.24. For the interactions, there was no interaction between time and group, BF10 = 0.25, while results were inconclusive regarding remoteness and group, BF10 = 1.70, time and remoteness, BF10 = 0.51, and time, remoteness, and group, BF10 = 0.42. Results were inconclusive regarding a change in positive-specific memory retrieval over time in the free-recall condition depending upon the age of the memory. To further examine remote positive-specific memories, correlation analyses were conducted (Pearson correlation coefficient) between the number of remote positive-specific memories and DASS depression scores at baseline, *r*(50) = 0.25, *p* = 0.09, and post-intervention, *r*(50) = 0.09, *p* = 0.54, revealing no significant correlations. Data were tested for outliers and linearity, meeting assumptions of the analysis.

### Changes in field perspective from pre- to post-intervention

A Bayesian repeated measures ANOVA was used for the total number of specific-field memories (across both remoteness conditions) with time (pre-and post-intervention) as a within-subjects factor, and group (LKM, colouring) as the between-subjects factor. An analysis of residuals confirmed the assumptions of linearity. Results were inconclusive regarding a main effect of time, BF10 = 1.90, and group, BF10 = 0.91, and regarding an interaction between time and group, BF10 = 0.57, contrary to our hypothesis.

A further Bayesian repeated measures ANOVA was performed on the total number of specific-field memories across both remoteness conditions using valence (positive, negative, neutral, and other) and time (pre- and post-intervention) as within-subjects factors, and group (LKM, colouring) as a between-subjects factor. Results were inconclusive regarding a main effect of time, BF10 = 0.71, and group, BF10 = 0.60, while there was a significant main effect of valence, BF10 = 4.84x10^+42^. Further, there was an interaction between time and valence, BF10 = 1.81x10^+43^, valence and group, BF10 = 4.42x10^+39^, and between time, valence, and group, BF10 = 4.84x10^+42^. However, there was no interaction between time and group, BF10 = 0.26. Bayesian post hoc paired t-tests revealed that there was a significant increase in positive-field memories (*t*(49) = -2.86, *p* = 0.006, *d* = -0.41, BF10 = 5.72); however, no reliable conclusion could be drawn regarding the retrieval of negative-field memories over time, (*t*(49) = 2.44, *p* = 0.02, *d* = 0.34, BF10 = 2.22), or retrieval of neutral-field memories over time, (*t*(49) = -1.27, *p* = 0.21, *d* = -0.18, BF10 = 0.33). Further, Bayesian and paired t-tests suggested no difference in retrieval of other-field memories over time, (*t*(49) = 0.57, *p* = 0.57, *d* = 0.08, BF10 = 3.71). Moreover, Bayesian independent t-tests indicated no reliable conclusion could be drawn regarding group differences in retrieval of positive-field memory retrieval over time, (*t*(48) = 0.93, *p* = 0.36, *d* = 0.26, BF10 = 0.40), or other-field memory over time, (*t*(48) = 1.76, *p* = 0.09, *d* = 0.50, BF10 = 0.99). Changes in negative-field, (*t*(48) = -0.54, *p* = 0.59, *d* = -0.15, BF10 = 0.32), and neutral-field memory retrieval, (*t*(48) = 0.54, *p* = 0.59, *d* = 0.15, BF10 = 0.32), were not found to differ depending upon group.

### Changes in specificity mediated by changes in self-discrepancies

The current study expected LKM to reduce self-discrepancies thus LKM was expected to impact features of AM retrieval via self-discrepancies. Mediation analyses were therefore performed below. Due to the small sample size for mediation analyses, bootstrapping was used with 5,000 bootstrapping samples and 95.0% confidence intervals for all the following mediation analyses.

A mediation analysis was conducted with the change in specificity as the outcome variable and change in focused self-discrepancies (from post- to post-self-discrepancy induction) as the predictor variable. Neither the LKM group, F(1, 23) = -1.52, p = 0.14, nor the colouring group, F(1, 23) = -0.63, p = 0.53 revealed significant mediation results.

Another mediation analysis was conducted with the change in specificity as the outcome variable and change in experienced self-discrepancies (from post- to post-self-discrepancy induction) as the predictor variable. Neither the LKM group, F(1, 23) = -0.71, p = 0.49, nor the colouring group, F(1, 23) = -0.17, p = 0.87 revealed significant mediation results.

### Changes in positive memory retrieval mediated by changes in self-discrepancies

A mediation analysis was conducted with the change in positive-specific memory retrieval as the outcome variable and change in focused self-discrepancies (from post- to post-self-discrepancy induction) as the predictor variable. Neither the LKM group, F(1, 23) = 2.39, p = 0.06, nor the colouring group, F(1, 23) = -0.22, p = 0.83 revealed significant mediation results; however, a trend for significance was observed in the LKM group.

Another mediation analysis was conducted with the change in positive-specific memory retrieval as the outcome variable and change in experienced self-discrepancies (from post- to post-self-discrepancy induction) as the predictor variable. Neither the LKM group, F(1, 23) = -1.87, p = 0.08, nor the colouring group, F(1, 23) = 0.96, p = 0.33 revealed significant mediation results; however, a trend for significance was observed in the LKM group.

### Changes in field perspective mediated by changes in self-discrepancies

A mediation analysis was conducted with the change in retrieval of specific-field memories as the outcome variable and change in focused self-discrepancies (from post- to post-self-discrepancy induction) as the predictor variable. Neither the LKM group, F(1, 23) = -0.31, p = 0.76, nor the colouring group, F(1, 23) = -1.00, p = 0.33 revealed significant mediation results.

A final mediation analysis was conducted with the change in retrieval of specific-field memories as the outcome variable and change in experienced self-discrepancies (from post- to post-self-discrepancy induction) as the predictor variable. Neither the LKM group, F(1, 23) = -0.32, p = 0.75, nor the colouring group, F(1, 23) = 0.02, p = 0.98 revealed significant mediation results.

## Discussion

Previous work has examined the impact of self-compassion on a variety of cognitive processes associated with depressogenic thinking. The current study extends this work to examine if a loving-kindness meditation can influence autobiographical memory patterns in remitted depression when negative thinking about the self is deliberately activated. The current study aimed to assess whether a brief LKM, in comparison to a colouring practice, would influence features of AM retrieval (specificity, remoteness, vantage perspective, valence) after inducing cognitive reactivity in remitted depression. We further aimed to identify whether baseline tendencies in a remitted depressed sample would show more remote as opposed to recent memory retrieval and whether positive-specific memories would be retrieved more remotely than recently.

The effects of both interventions on depressive symptoms and characteristics were assessed. Changes in negative affect (DASS), depression (DASS depression), and self-compassion (SCS) over time were inconclusive and there were no significant group differences in changes in negative affect or depression. However, results regarding group differences in changes in self-compassion were inconclusive. Alternatively, while rumination (RRS), experienced self-discrepancies, and focused self-discrepancies did improve over time, results were inconclusive regarding differences in improvement between groups.

Findings supported our hypothesis that total memory specificity would increase over time in the LKM group only. However, an anomalous finding was also observed where rumination positively correlated with specificity and both specificity and rumination improved over time. This finding might be due to the sample desiring to perform well on the AMT possibly as a result of the Covid-19 pandemic. For example, it might be that due to drastic pandemic-related changes in teaching methodology at the university, participants had higher levels of perfectionism that resulted in better performance (i.e. specificity) on the AMT while negative affect remained higher and self-compassion remained lower. While this is speculative, as measures of perfectionism were not obtained, the relationship between rumination and specificity might vary depending upon personality factors which could be a further consideration in future studies. Despite this surprising correlation, specificity at baseline was low in this sample overall, consistent with previous literature [18], highlighting the enduring tendency of OGM in remitted depression. Contrary to predictions, however, results were inconclusive regarding an increase in specificity in the free-recall condition depending upon the age of the memory. Considering the average age of memories in the free-recall condition was only one month to one year, there was not a greater opportunity to improve specificity in remote memories. Further results were inconclusive regarding the influence of LKM on the specificity of remote memories in the remote condition, where the DMS was between 13 months to 5 years. This could suggest that remote specificity is more difficult to impact and might require a longer intervention duration.

Our hypothesis that the LKM group would retrieve more positive-specific memories over time in comparison to the colouring group was not supported given results were inconclusive. While LKM might have altered the way events were perceived, such that memories previously perceived as neutral could have been shifted to a positive valence, the colouring practice might have distracted participants from more negative memories, making positive memories more readily available. Further analyses were inconclusive regarding greater increase in recent positive-specific than remote positive-specific memories in the LKM group (free-recall condition). Although this does not support our hypothesis, given the LKM group did not show a baseline tendency to retrieve more remote positive-specific memories, this finding is not surprising.

Further, results were inconclusive regarding the retrieval of more specific-field memories over time and regarding a group interaction, contrary to predictions. Moreover, it was unclear whether an increased retrieval of positive-field memories depended upon group. Given literature has demonstrated a tendency for healthy samples to use field perspective for positive memories and observer perspective for negative memories [27, 28], the increased use of field perspective for only positive memories that was seen across groups might be more beneficial than increasing field memories regardless of valence. For example, increasing the use of field perspective for negative memories could be expected to increase the emotionality of the memories (e.g. pain) [23].

At baseline, it was hypothesised that more remote memories would be retrieved than recent memories given a focus on self-discrepancies was activated; however, this prediction was not supported. Although cognitive reactivity was successfully induced, it might be that focused self-discrepancies were not sufficiently high to necessitate retrieval of remote memories to distance oneself from recent memories that painfully highlight current discrepancies. While the current study did not compare remoteness tendencies of the remitted depressed sample to that of a never-depressed sample, our remitted sample did reveal a similar average age of memories to that found in dysphoria [20]. Further, we expected positive-specific memories would be retrieved from a more remote as opposed to recent period but differences in remoteness were not found. Although previous literature has demonstrated self-reported depression severity predicted positive memory remoteness [21], remote positive-specific memories were not found to correlate with depression scores (DASS depression) in the current study. It might be that more severe depressive symptomatology (i.e. currently depressed samples) would reveal this relationship.

A trend in significance for self-discrepancies to mediate the changes in retrieval of positive-specific memories was revealed in the LKM group only. However, changes in self-discrepancies were not found to mediate changes in specificity or retrieval of specific-field memories in the LKM group contrary to expectations. Given the number of Bayes Factors that indicated inconclusive data however, it could be that data are too uncertain to draw conclusions about self-discrepancies mediating changes in specificity or specific-field memories. At this point, it is unclear what mechanisms of LKM influenced changes in features of AM retrieval. Thus, the next steps in line with this research would be to focus on elucidating mechanisms of change.

Our findings have implications for developing treatments to alter features of AM retrieval in remitted depression and reducing a cognitive vulnerability to depression. The current study demonstrated significant improvement in specificity, valence, and vantage perspective, whereas previous treatments have largely focused on single or dual features of AM retrieval in interventions. For example, Memory Specificity Training (MEST) explicitly teaches participants in group sessions [85] or via computerized tasks [86] to retrieve specific memories using positive and negative cues, yielding significant improvement in specificity. Alternatively, Mindfulness-Based Cognitive Therapy (MBCT) has been used to target both OGM and remoteness [76]. In comparison to a treatment-as-usual control group, MBCT reduced OGM in a remitted depressed sample. Moreover, the MBCT group retrieved memories from a more recent period in comparison to the controls. Critically, in the current study, both groups retrieved an increase in recent memories at follow-up, indicating that it was not only the MBCT intervention that influenced remoteness. Our findings suggest, however, that LKM might provide a broadly accessible treatment to influence specificity, valence, and vantage perspective, while a colouring practice might also influence AM valence and vantage perspective. Remoteness might yet be impacted if examined in a sample with a baseline tendency to retrieve more remote memories. Further examination of the influence of LKM on features of AM retrieval would extend research on self-compassion and its underlying mechanisms as self-compassion has not been investigated in the context of AM retrieval prior to the current study.

### Limitations and future research

There were limitations to the current study that should be considered when interpreting our results. Our sample was exclusively drawn from a university student population, decreasing the generalizability of our findings to clinical settings. However, it should be pointed out that depression is very prevalent in this population [87], making it a very important group to study. Further, all participants met the criteria for remitted depression according to the DSM-V. Nonetheless, it is important to test whether our findings replicate in clinical samples with current and remitted depression. Moreover, data were not collected regarding the date of participants’ most recent depressive episode. This might be a factor that influences intervention success and therefore needs to be examined in future research. Further, participants were not asked to refrain from beginning any new form of individual psychotherapy or meditation practice during the time of the study. Future studies would benefit from setting these parameters. Moreover, given some of our baseline variables compared for group equivalence revealed Bayes factors between 0.33 and 3.00, indicating uncertainty in the data, it will be important to collect more data to gain further clarity. Importantly, however, as the Bayes factor deviates from 1, more support is collected for *H*_*0*_ or *H*_*1*_ [88], and our Bayes factors were closer to 0.33 on the classification table, suggesting there was more support for group baseline equivalence.

Another limitation was the choice of procedure for the AMT. Although previous studies have successfully employed written versions of the task [68], a 60 second time constraint on writing might not have provided a sufficient amount of time for memory retrieval. Another important metric with the AMT is the number of details retrieved. With the use of a written procedure, writing could impact participants’ ability to retrieve more detail. However, the current study did not ask participants to retrieve as many details as possible and this was not a focus of analysis. Memory detail is an important factor to investigate however [89], and future research could aim to replicate results with no time constraint on the AMT, allowing for retrieval of more detail. This methodological alteration could further allow for examination of self-compassion representations in the memory content.

A further limitation was our definition of remote memories as being over one year old. Previous literature has applied varying boundaries of remoteness from one [20, 21] to ten years [90], yielding incomparable results. While younger participants would not be suited to abide by a ten-year remoteness parameter for example, this field could improve methodological heterogeneity by examining boundary differences. Further, due to a technical issue, cue words were not consistently randomised between remoteness conditions which will be important to ensure in future studies. Additionally, the cue-word paradigm that was employed (AMT) might not be representative of daily experiences of memory retrieval [91]. It would be beneficial to assess whether our findings replicate for AM elicited in everyday circumstances such as within social interactions. Furthermore, it is important to address the possibility that results could be due to a practice effect. It could be that all participants underperformed on the AMT at baseline due to the novelty of the task and that post-intervention, participants became more confident or comfortable with the task. However, our data suggest that a practice effect did not account for the results as a significant increase in specificity was only demonstrated in the LKM group, and the LKM group demonstrated larger, non-significant increases in retrieval of positive memories and field memories in comparison to the colouring group.

In conclusion, findings in the current study provide initial insights into the effects of LKM on impaired features of AM retrieval in remitted depression. LKM has been identified as a potential intervention to increase retrieval of specific, positive, and field memories in remitted depression. The current study further shows that LKM can act as a buffer for the effects of AM when cognitive reactivity is induced by a self-discrepancy task. It will be important to compare the effectiveness of LKM and MEST for example. Current models, such as MEST, train specificity with the aim that when stress is encountered, increased specificity will remain; however, MEST does not address the issue of cognitive reactivity and does not influence emotions which can generate memories. Further, future studies including fMRI would allow us to understand the neural basis of self-compassion and the neural effects of LKM on AM retrieval. Future research should also examine whether LKM can influence tryptophan as well as other biological factors, such as cortisol, and the impact of influencing these factors on features of AM retrieval. Our findings necessitate research in the field of self-compassion practices to converge with AM retrieval in current and remitted depression.

### Consent to participate

Digital informed, written consent was obtained from all individual participants included in the study. Participants were informed that participation in the study was voluntary.

### Consent to publish

Participants provided informed, written consent regarding publishing their data.
